# Small teeth, big opportunities: strengthening oral health communication in early years settings

**DOI:** 10.1186/s13690-026-01927-x

**Published:** 2026-05-18

**Authors:** Eleanor Forshaw, Karen Vinall-Collier, Amrit Chauhan, Jayne Purdy, Sumiah Alnofal, Kara Gray-Burrows, Peter F. Day

**Affiliations:** 1 University of Leeds, Leeds, UK; 2 Community Dental Service, University of Leeds, Bradford District Care NHS Foundation Trust, Leeds, Bradford, UK

**Keywords:** Oral Health [MeSH], Dental Caries/prevention & control [MeSH], Toothbrushing/psychology [MeSH], Parents/psychology [MeSH], Caregivers/education [MeSH], Nurseries, Infant [MeSH], Health Communication/methods [MeSH], Qualitative Research [MeSH], Early years settings, Thematic analysis, Health inequalities

## Abstract

**Background:**

Dental caries remains a significant public health issue across the UK. Early years settings, such as nurseries and preschool playgroups, provide a unique opportunity to promote oral health, yet implementation of oral health interventions varies widely. This study explored early years professionals’ and parents’ views on oral health activities and conversations undertaken within early years settings in a socioeconomically and ethnically diverse English city.

**Methods:**

Semi-structured qualitative interviews and focus groups were undertaken with early years professionals and parents of children aged 0–4 years old. Topic guides were informed by the COM-B model. Interviews and focus groups were audio-recorded, transcribed, anonymised, and analysed using reflexive thematic analysis in NVivo.

**Results:**

Sixteen early years professionals and fourteen parents participated. Three key themes were identified. (1) Collaboration between early years settings and home: parents valued the authority of early years staff in encouraging oral health routines, while staff highlighted the importance of trust and clear communication to support shared responsibility. (2) Embedding oral health in practice: staff were motivated to promote oral health and often created innovative activities, such as songs, role play, and links to healthy eating, which families reported as beneficial. (3) Sustaining oral health beyond the setting: parents acknowledged that maintaining routines at home could be difficult, but described how structured practices within early years settings, alongside peer influence and regular reinforcement, supported children’s engagement. Both groups recognised the value of early years settings in supporting oral health but highlighted some inconsistency in delivery and uncertainty around roles. While structured routines were seen as effective, sustaining behaviours beyond the settings remained a challenge.

**Conclusion:**

Early years settings have strong potential to promote children’s oral health. Building on existing creativity, consistent training, clear communication, and stronger links with dental services could help ensure these efforts are sustained and equitable.

**Table:** 

Text box 1. Contributions to the literature	
• Evidence on how systemic factors within early years settings shape oral health promotion remains limited; this study highlights structural influences beyond individual staff practice.	
• Structural inequalities and restricted access to NHS dental services create persistent barriers to maintaining oral health at home; addressing these constraints is essential to improve equity.	
• Sensory sensitivities act as a universal barrier to oral health routines in early childhood, affecting children with and without SEND diagnoses, and are often harder to manage where experiences are framed through SEND.	
• Coordination between early years settings, health visiting, and dental services remains limited, reducing consistent preventive support.	

## Background

Despite the importance of oral health during early childhood, a significant percentage of children across the United Kingdom (UK) suffer from preventable oral diseases [1]; with the most common being dental caries. The impacts are profound, affecting both the child and their family’s quality of life and daily activities [2]. Caries is the leading cause of hospital admissions among children aged 5–9 years old, with over 30,000 episodes of tooth extractions due to dental caries carried out for 0–19-year-olds in England per annum [3]. Current literature affirms the importance of early preventive measures and optimal oral health habits, which include twice daily toothbrushing with a fluoride toothpaste, maintaining a low sugar diet, and regular dental check-ups [4]. With a strategic shift towards prioritising preventive care reflected in the National Health Service (NHS) Long Term Plan [5], the inequalities in healthcare access that were both exposed and exacerbated during the COVID-19 pandemic have prompted an emphasis on improving children’s health services, including oral health [6].

Within this landscape, the role of early years settings in promoting health and development is already identified in national policy. The Early Years Foundation Stage statutory framework requires providers to *“promote good oral health”* as part of their safeguarding and welfare responsibilities [7]. Supporting guidance from the Department for Education aligns with preventive best practice, and recommends reducing free sugars, encouraging twice-daily fluoride use, and promoting early dental attendance [8]. This reflects national priorities. The recent government-funded, national roll-out of supervised toothbrushing programmes signals a renewed policy emphasis on tackling childhood caries at a population level [9], ands aims to target children aged 3–5 years old living in the most deprived areas of England. It is important to note that at the time of data collection for this research, these statutory requirements and national programmes were not yet in place, and oral health promotion remained encouraged rather than compulsory.

These commitments align with wider government priorities around school readiness and attendance, which reinforce the expectation that early childhood services support children’s holistic development [10, 11]. School readiness has become a central policy priority, reflecting evidence that inequalities in health and development during the early years strongly influence later educational achievement and life chances [12]. Persistent absence in the early years is also increasingly recognised as a barrier to long-term outcomes, prompting the government’s Opportunity for All mission to focus on improving attendance and tackling disadvantage from the earliest stages [12]. Oral health is a critical foundation of this, with dental pain, disrupted sleep, and treatment-related absences all compromising a children’s readiness to learn and capacity to thrive in educational settings [2, 13]. Evidence from initiatives such as Sure Start demonstrates the value of early years programmes in addressing these inequalities and supporting family wellbeing, while recent Child of the North reports have emphasised the potential of schools and early years services to act as health hubs within local communities [14–16].

Bradford provides an important context for this research. As a city positioned within the north of England, almost half of its electoral wards are among the most deprived 10% in the country [17]. Oral health inequalities are marked; the prevalence of dental caries among five-year-olds is 36%, considerably higher than both the national average of 23%, and the 13.7% observed in the least deprived area of the country [18]. This underscores the significant socioeconomic challenges that shape children’s health and development in this area and highlights the importance of understanding how early years settings can support oral health promotion in contexts of high need and multiple family priorities.

Despite policy commitments, oral health has received comparatively little attention within early years practice. Parents have previously reported that nurseries are often not perceived as environments for oral health promotion, with limited visibility of oral health activities and uncertainty about staff roles in supporting children’s oral health [19]. Where programmes *are* introduced, delivery is highly variable between settings and across local authorities, reflecting differences in resources, priorities, and capacity [20]. This inconsistency has created a lack of clarity about how oral health promotion is best embedded within early years provision, and how such activities are understood and experienced by families and professionals [19].

This study therefore aimed to explore early years professionals’ and parents’ views on, and experiences of, supportive oral health conversations and related activities within early years settings.

## Methods

This qualitative study was conducted as part of the Better Start Bradford initiative, which aims to improve the health and well-being of young children (aged 0–4 years old) and their families in three deprived areas of Bradford; Bowling and Barkerend, Bradford Moor and Little Horton.

### Sample

The study included early years professionals and parents of young children attending early years settings within the Better Start Bradford area. Sampling and recruitment procedures differed across participant groups and are described separately below.

Early years settings located within the Better Start Bradford programme area were identified through the programme’s existing networks. A member of the Better Start Bradford team initially contacted 31 settings to seek permission to share their details with the research team. The 6 settings that agreed were subsequently contacted by members of the research team, who provided further information about the study and worked with setting managers to organise data collection sessions.

Within participating settings, early years professionals were eligible to take part if they were employed within the setting and involved in oral health activities or conversations with parents. Potential participants were identified with the support of senior leadership teams, who circulated participant information sheets to staff. Interested staff members were given at least three days to consider participation before providing written consent.

Parents were eligible to participate if they had at least one child aged 0–4 years old attending a participating early years setting. Parents were recruited indirectly through early years settings. Recruitment was supported through the display and distribution of study posters and participant information sheets within settings. In some cases, members of the research team attended settings to provide posters to staff to support awareness of the study. Parents who were interested in taking part contacted the research team directly. Parent recruitment therefore followed a convenience, opt-in approach and was mediated by early years settings as gatekeepers.

The researchers did not have prior relationships with any participants involved in data collection.

### Data collection

Data were collected through a combination of semi-structured interviews and focus groups with early years professionals and parents, by KVC and JP. The topic guides for all sessions were informed by the COM-B model, which conceptualises behaviour as the result of interacting Capability, Opportunity and Motivation factors [21]. This was applied here to structure the exploration of oral health activities and conversations within early years settings, with the guides used flexibly to ensure coverage of these domains while allowing participants to raise additional issues.

Interviews and focus groups varied in duration, reflecting participants’ availability and the pragmatic constraints of conducting research with early years professionals and parents. Focus groups ranged from approximately 32 to 53 min, individual interviews with parents from 15 to 22 min, and interviews with early years staff from 17 to 50 min. Shorter interviews were typically more focused, often centring on specific routines or challenges, while longer interviews and focus groups allowed for broader reflection and interaction.

For the parent sessions, two focus groups took place in person. Although focus groups were initially planned for all parent participants, time constraints after childcare sessions limited attendance, so individual interviews were introduced during recruitment to maximise participation. These were primarily conducted by telephone to accommodate participants’ preference and availability. Only one parent focus group required in-situ translation, which was provided by a bilingual member of early years staff. The staff focus groups and interviews all took place via Zoom, and none required translation. No sessions were repeated.

Data collection methods generated different forms of data. Focus groups produced interactional accounts, enabling participants to build on one another’s experiences, whereas individual interviews elicited more personal, routine-based narratives. These data were treated as analytically complementary rather than equivalent, and reflexive thematic analysis attended to both content and context when developing themes. Analytic depth was achieved across the dataset through convergence of patterns across methods and participant groups, rather than through the duration of individual interviews.

All sessions were audio-recorded with permission and transcribed verbatim by a professional transcription service. Field notes were taken during and after sessions to capture contextual observations, but transcripts were not returned to participants for checking, reflecting both resource constraints and a desire to minimise additional burden on busy early years staff and parents. Data collection continued until the research team agreed that sufficient depth and diversity of perspectives had been obtained to address the study aims.

### Positionality

This study was conducted within a critical realist ontology, recognising participants’ accounts as reflecting real experiences while also shaped by social and contextual factors. The research team comprised researchers based within a dental public health research group, with diverse disciplinary backgrounds including public health, psychology, and qualitative research, alongside expertise in paediatric oral health and behaviour change.

Data collection was undertaken by KVC and JP, who were not previously known to participants, reducing potential influence from pre-existing relationships. The team’s positioning within dental public health, however, may have shaped how parents experienced the interviews, and there is a risk that participants felt pressure to provide socially desirable responses when discussing oral health behaviours. To mitigate this, interviewers emphasised that there were no right or wrong answers, encouraging discussion of challenges, compromises, and everyday decision-making.

The team’s professional commitment to improving oral health may also have influenced the framing of questions and interpretation of findings. Reflexive discussions were therefore held throughout analysis to critically consider these influences and to ensure that themes remained grounded in participants’ accounts and situated within their wider social and material contexts rather than professional assumptions.

### Analysis

The final transcripts were reviewed against the original audio for accuracy, anonymised, and securely stored. Data were analysed using reflexive thematic analysis following Braun and Clarke’s [22, 23] six-phase process: (1) familiarisation with the data; (2) systematic coding relevant to the study’s objectives; (3) initial theme development from coded data; (4) reviewing and refining themes; (5) defining and naming themes; and (6) producing the final narrative account.

While the topic guides were informed by the COM-B model, analysis itself was conducted inductively, with coding and theme development not structured around COM-B constructs. Themes were generated through a reflexive, interpretative engagement with participants’ accounts rather than mapped onto a predetermined theoretical framework. In line with Braun and Clarke’s conceptualisation of reflexive thematic analysis, prior theoretical knowledge was understood as shaping the research process but not determining analytic outcomes; reflexivity was therefore used to critically engage with these influences rather than treat the analysis as theory-free.

Coding and theme development were iterative, moving between data and analysis as interpretations developed, and NVivo was used to support data organisation and retrieval. Data from parents and early years professionals were initially analysed separately to account for the distinct perspectives of each group. This enabled identification of patterns within each cohort before considering points of convergence and divergence across both datasets. While organisational dynamics differ across early years settings, the analysis focused on identifying cross-cutting mechanisms shaping oral health practices across early years contexts rather than comparing experiences by setting type.

Preliminary themes were initially proposed by KVC, and these were subsequently developed further by EF, AC, KG-B and PD (with academic backgrounds in qualitative research, dentistry, and psychology), with final themes agreed collaboratively. In line with Braun and Clarke’s reflexive thematic analysis, analytic validity was not assessed through participant verification of transcripts or themes; accordingly, participants from the original study were not invited to provide feedback on the findings. Credibility was instead supported through reflexive, team-based analysis involving researchers from diverse disciplinary backgrounds. Interpretations were iteratively discussed within the team, and the completed analysis was reviewed by colleagues outside the core research team, including practitioners not involved in dental research, to provide critical challenge and assess the plausibility and clarity of interpretations.

### Ethical considerations

Ethical approval for the study was obtained from the University of Leeds Research Ethics Committee (DREC reference: 220120/PD/296). Participants were asked to read an information sheet and give both written and verbal consent before data collection commenced. This information outlined the maintenance of their confidentiality, and the anonymisation and storage of their data.

## Findings

Sixteen early years professionals and fourteen parents participated in the study. Staff members were recruited from six early years settings across multiple wards of Bradford, comprising one childminder, three day nurseries, one preschool playgroup, and one primary school with an attached nursery. Staff took part in two focus groups (*n* = 8) and eight individual interviews (*n* = 8). All staff participants were female.

Parents were recruited via early years staff members and participated in two focus groups (*n* = 9) and five individual interviews (*n* = 5). Most parent participants were mothers, with one father, and the sample reflected Bradford’s ethnic diversity, including Pakistani, Bangladeshi, African, White, and mixed ethnic backgrounds. The parents who took part had children aged between 1 and 3 years old, both girls and boys, attending the included early years settings.

Three overarching themes were developed, reflecting the perspectives of both groups: (1) Collaboration Between Early Years Settings and Home, (2) Embedding Oral Health in Practice, and (3) Supporting Continuity of Oral Health at Home. Each theme comprised several subthemes as outlined in Table 1. Together, they capture early years professional’s and parents’ views on oral health activities undertaken within early years setting, and how this translates to sustaining oral health habits at home.

**Table 1:** Themes and subthemes from qualitative analysis of oral health activities in early years settings

Theme	Subthemes
1. Collaboration Between Early Years Settings and Home	1.a. Improving communication channels
	1.b. Lost in translation
	1.c. Who “takes care” of oral health?
	1.d. Making collaboration work
2. Oral Health in Practice	2.a. Knowledge gaps
	2.b. Structural barriers
	2.c. Supporting families with Special Educational Needs and/or Disabilities (SEND).
	2.d. Creative solutions in practice
3. Sustaining Oral Health Beyond the Setting	3.a. When oral health routines break down
	3.b. Reinforcement through shared responsibility
	3.c. Disconnections in the pathway of care

*SEND* Special Educational Needs and/or Disabilities

### Theme 1: Collaboration between early years settings and home

This theme captured how oral health activities carried out in early years settings were shaped by the quality of collaboration with families. Parents and staff both described the opportunities and tensions in working together, with communication practices, clarity of responsibility, and the presence or absence of trust all influencing how oral health conversations were understood and acted on.

#### Improving communication channels

Parents frequently reported minimal awareness of oral health activities within early years settings. When parents were able to share their understanding of what was being carried out in these settings, their knowledge was generally limited to supervised toothbrushing programmes or healthy eating:

“No not talked about very much. They’ll, they’ll message this WhatsApp group for all the parents on the, for the school. And then they’ll just occasionally update that” (Parent, Interview).

While most of the staff members were not initially aware of this gap in understanding, it did become clear to them throughout the interview process and during the shared focus group:

“Last month, we did a whole month about healthy body… we did some activities with healthy teeth also, so I don’t know if any of you would have received pictures of them.” (Playgroup Staff Member, Focus Group).

“Because we have got it on the newsletter recently saying that we are doing brush teeth, but if still are parents are unaware, it just seems like the message has not gone through” (Playgroup Staff Member, Focus Group).

Together with parents’ earlier descriptions of oral health communication, or lack thereof, these accounts suggest a disconnect between staff intentions and parental awareness. While staff described sustained activity related to oral health, this was not consistently recognised by parents as oral health communication, indicating a breakdown in communication that limited parental engagement rather than an absence of activity. This disconnect was reflected in the channels through which information was typically shared. Much of the information being shared by early years settings was one-directional, usually occurred through WhatsApp group announcements or newsletters, which parents found easy to overlook. In settings where these digital broadcasting methods were not being utilised, one parent explained that oral health was *“not talked about very much…unless [she] asked something”.* Staff accounts supported this, indicating that while conversations did take place, they were often reactive rather than planned or systematic:

“I think we just spontaneously will stop and have a conversation with our parents… one-to-one, on the way in and out of nursery.” (Primary School Nursery Staff Member, Focus Group).

Taken together, these descriptions suggest that parents who did not actively prompt staff, or who had less opportunity at drop-off/collection, were less likely to receive information. Where this direct engagement with parents was constrained, some staff channelled conversations through children during everyday activities:

“We do talk about, like we’ll ask the children, ‘oh what have you had for breakfast this morning?’ (PreSchool Playgroup Staff Member, Focus Group).

“But it’ll be not with their parents, it’ll be with a child yeah…. You sit down and have a chat and we have circle time as well.” (PreSchool Playgroup Staff Member, Focus Group).

This indirect, child-mediated route made oral health visible within the setting, but given the children’s age, risked variable recall and left some parents less directly informed.

#### Lost in translation

Language and literacy were key factors shaping how oral health information was communicated and received. In several settings, staff described how written resources such as newsletters or advice slips were ineffective for parents with limited English or lower literacy levels. This meant that important guidance, for example following fluoride varnish application, was not always read or acted on:

“Sometimes as well following fluoride, if it’s just an advice piece of paper… lots and lots of our parents, English is an additional language… so they don’t even read pieces of paper that are given to them.” (Primary School Nursery Staff Member, Focus Group).

This setting addressed this by utilising bilingual staff who could translate directly during conversations with parents. While these personal exchanges were dependent on the availability of particular staff members, they were seen as very effective:

“[We have] lots of languages that are spoken within our nursery setting that it didn’t feel like language barrier was a problem in that situation” (Primary School Nursery Staff Member, Focus Group).

The settings that were able to do this also relied less on written resources, and often utilised activities or visual tools instead. A smaller number of participants also pointed to digital exclusion, noting that some families were unable to access app-based updates:

“We do have an app and everybody, mostly everybody, [does] access. We do have some parents who have got little bit difficulty with technology, so it’s more of verbal feedback to them.” (PreSchool Playgroup Staff Member, Focus Group).

These accounts suggest that reliance on either physical or digital written communication risked excluding families with additional language or literacy needs. While oral health messages could be successfully communicated in face-to-face interactions, these narratives indicate that families who were not engaged in these conversations may miss out altogether.

#### Who ‘takes care’ of oral health?

Parents frequently described early years staff as having greater authority than themselves. Many explained that their children were more likely to follow oral health routines when directed by staff, immediately positioning the early years settings as an important influence on children’s habits:

“As a parent that I notice with, with both of my kids, if I tell them something they don’t really listen…. But if it comes from the teacher they do.” (Parent, Focus Group).

Despite this, multiple early years staff showed concern about capacity and role boundaries. While supportive of oral health promotion, they felt that responsibility ultimately lay with families, and questioned whether it was appropriate to absorb this into their already stretched workload:

“You’ve got your role of education to the children, but now we’re also saying, ‘Can you just educate the parents as well while you’re at it?’ [Laughs]. It’s quite a big ask, isn’t it?” (Primary School Nursery Staff Member, Focus Group).

These individuals also indicated that when parents were aware of supervised toothbrushing taking place in the setting, some felt less of a requirement to supervise toothbrushing at home. Staff worried this created a false sense of reassurance, with parents assuming the setting’s efforts were sufficient:

“Parents got to the point where it demotivated them and it deskilled them because they said, ‘Oh, you’re doing it at school, you don’t need to do it at home’… and that really concerned me because actually…it needs to be a parental role, doesn’t it? It’s like a skill for life.” (Day Nursery Manager, Interview).

These conversations highlighted the tension between parents’ reliance on nurseries as trusted sources of authority, and staff’s recognition that oral health needed to remain a shared responsibility.

#### Making collaboration work

Staff suggested that collaboration with families would feel easier if oral health was built into the curriculum. They felt that having oral health formally included would create a shared sense of responsibility; staff would feel accountable for delivering activities, and parents could see oral health positioned alongside other areas of learning:

“If it’s there in black and white that we have to be touching on it in some sort of respects, we’d definitely pay more attention to making sure that the activities were in place… once it’s in the curriculum, then I definitely think that we could do a bit more” (Primary School Nursery Staff Member, Focus Group).

At the time of data collection, oral health promotion was encouraged but not compulsory; participants’ calls for curriculum inclusion therefore anticipated policy changes that have since been formalised within the Early Years Foundation Stage statutory framework. As an example of where this was already working successfully, both parents and staff frequently referred to toilet training and healthy eating as models of joint working. The staff described them as a structured, individualised process, underpinned by specific guidance and regular communication with families, and suggested that oral health could benefit from a similar approach:

“[When] parents access it then they get an individual phone call, we drop off a bag which we give them, you know, pants, knickers, prompt sheets, stickers, a chart, you know … and we will do a similar thing with the toothbrushing … It has to be a very individualised approach really.” (Day Nursery Manager, Interview).

”That links on a daily basis in terms of … that healthy eating policy. That winds into the curriculum, so the children understand why … we’re having what we’re having for snack … so it really focuses the children on thinking about their oral health in kind of an educational way.” (Day Nursery Manager, Interview).

Both groups of participants also reflected on how these principles could be actively extended into practice for oral health. Parents and staff proposed that integrating oral health into the same digital feedback systems already used for everyday updates would help ensure that information was regular, routine, and accessible:

“App, I think, because I always check app, that’s why.” (Parent, Focus Group).

“…probably putting it on the app, on the daily feedback, would be a good option…” (Day Nursery Staff Member, Focus Group).

This suggestion highlighted a desire for oral health to be embedded within existing communication structures, rather than treated as an additional or one-off message, making collaboration feel more manageable for both staff and families. Staff also noted that communication often became concentrated around specific initiatives, such as fluoride varnish visits, and risked losing momentum once these ended. They emphasised that collaboration was more sustainable when supported by smaller, ongoing conversations in between these larger events:

“we do like a big thing when the fluoride [varnish team] are coming in… but we’re all probably a little bit guilty then of letting it just sort of slip off… it’s the in-between parts that we need to look at developing… in a way that’s also manageable for a lot of us that have quite big workloads as well on top.” (Primary School Nursery Staff Member, Focus Group).

This account illustrates how the concept of collaboration was not only about the content of oral health messages but also their consistency, with staff recognising that parents were more likely to stay engaged when oral health was woven into routine contact rather than dependent on occasional campaigns.

Finally, staff members reflected that even when systems and structures were in place, parents were more receptive when advice came through informal, ongoing relationships. A clear sentiment was that trust could turn oral health from a task-based message into a conversation that parents felt comfortable engaging with:

“It’s all to do with how you are with the parents. I’ve got a great partnership with the parents, so if I’m going to encourage oral [health] or whatever healthy eating … they’re happy to participate, you know that they’ll be happy to support me on that and support their own children.” (Childminder, Interview).

“Opens up really informal but important conversations… if they’ve got your trust… the conversation doesn’t seem as hard.” (Primary School Nursery Staff Member, Focus Group).

Taken together, these narratives suggest that both parents and staff see the potential for strong collaboration when oral health is embedded into the curriculum, reinforced through routine and structured communication, and approached within relationships built on trust:

### Theme 2: Oral health in practice

While the first theme highlighted the dynamics of collaboration between parents and early years staff, participants also reflected on how oral health activities were delivered within early years settings themselves. This theme focuses on staff perspectives, exploring how knowledge, training, and practical challenges shaped their ability to embed oral health into daily practice.

#### Knowledge gaps

Many staff members reported feeling undertrained and under-supported in delivering oral health advice. They described lacking confidence in giving specific guidance and being uncertain about where to access reliable information. When asked whether oral health training would help, one Early Years staff member said:

“If she comes to me and says to me that my child is getting this stain [on their teeth] … I won’t be doing any suggestion without any knowledge of this, so maybe a little short training on that … on the basic things of oral health would be something.” (PreSchool Playgroup Staff Member, Focus Group).

This lack of confidence was compounded by concern about how advice might be received. Multiple staff members explained that they sometimes avoided raising oral health issues for fear of sounding judgmental or making parents feel criticised.

“… if we notice children have poor teeth… it is a bit sensitive to address that, cause you know, you want to not offend the parent and make the parent feel that they’re being judged.” (Primary School Nursery Staff Member, Focus Group).

Within this lack of training, the most significant gap identified related to supervised toothbrushing. While staff often framed independent toothbrushing as a developmental goal, this contrasts with UK guidance in place at the time of data collection, which recommended adult supervision or assistance with toothbrushing until at least age seven, and which remains unchanged in current guidance:

“And we’ve been showing the… how to do, how to do it themselves as well.” (PreSchool Playgroup Staff Member, Focus Group).

Parents often interpreted this practice as validation that independent toothbrushing was appropriate, reinforcing habits that conflicted with current recommendations.

“Because nursery’s teaching her herself so she wants to do it herself.” (Parent, Interview).

These accounts highlight that while staff were motivated to promote oral health, gaps in training and confidence may be limiting their ability to provide accurate, consistent advice. The example of independent toothbrushing illustrated how these gaps could have unintended consequences, with practices in the setting shaping what families did at home rather than explaining these differences to children and their parents.

#### Structural barriers

Staff also reflected on how structural constraints limited their ability to deliver oral health activities. COVID-19 was described as a major turning point, with many initiatives suspended during the pandemic and not fully reinstated. External partners who had previously provided demonstrations, resources, and fluoride varnish visits were no longer present, reducing both the visibility of oral health and opportunities to engage parents directly:

“We had the Better Start Agency… they were able to talk to parents and engage with the children … that was really well received. And then normally we have fluoride varnishing… that was really useful. But obviously, that has stopped now because of Covid.” (Primary School Nursery Staff Member, Focus Group).

Alongside the pandemic, staff highlighted how funding cuts had reduced capacity for oral health promotion. Activities that had once been routine, such as parent workshops, had been discontinued due to resource constraints, whilst other community initiatives like “*bin the bottle*” were temporarily halted:

“We would have workshops. We used to have them in the children’s centre, which it’s been cut now, we don’t have that facility anymore.” (Primary School Nursery Staff Member, Focus Group).

“But lots of that funding has gone for us … to have those events, to come in to buy provisions and to kind of get people involved.” (Primary School Nursery Staff Member, Focus Group).

These accounts illustrate how structural factors, including the pandemic and service defunding, curtailed the range and consistency of oral health activities available in settings and impacted regular delivery. Many of these staff members were passionate about the value of such initiatives in supporting both children and parents but acknowledged that capacity and cost pressures had made it difficult to sustain them.

#### Supporting families with Special Educational Needs and/or Disabilities (SEND)

Across participant groups, sensory challenges were described as a common barrier to establishing oral health routines in early childhood. Parents frequently discussed aversion to the taste and texture of toothpaste, the texture of food, and the sensation of brushing as factors that disrupted both toothbrushing and eating practices. These difficulties were reported by almost all parents interviewed and were not limited to children with a formal SEND diagnosis.

Staff and parents did, however, describe these challenges as more difficult to manage when children’s sensory experiences were framed through SEND. In these cases, resistance to oral health activities was often more intense, progress towards routines was slower, and strategies that worked for other children were less effective or required sustained adaptation:

“…he gets really aggressive for toothbrushing, so he does not do toothbrushing at home, he is only [using a toothbrush] when he comes to nursery… He just recently started to just put the toothbrush in the mouth, he is still not brushing it, but we are still encouraging.” (PreSchool Playgroup Staff Member, Focus Group).

”…he doesn’t like the touch of water or anything, because of his sensitive needs, so I think this… it’s more of a sensory thing than anything else.” (Parent, Focus Group).

Given the young age of the children, these accounts often reflected everyday identification of additional needs by parents and staff rather than confirmed diagnoses. Sensory difficulties were recognised through routine interactions and shaped how oral health practices were negotiated at home and within early years settings.

Staff noted that children whose sensory experiences were understood within a SEND framework often required more individualised approaches, such as adapted toothbrushes or altered routines. Despite this, staff also described limited guidance on how to support families in managing these challenges consistently across settings and home:

“I personally think there should be a little bit, a bit more research, a bit more input in children with special education needs, and I do feel there is no help that if a child has got sensitive needs” (PreSchool Playgroup Staff Member, Focus Group).

While these accounts indicated that sensory barriers are a common feature of early childhood oral health routines, they become more complex and harder to resolve when children’s experiences are framed through SEND.

#### Creative solutions in practice

Despite minimal formal support or structured resources, many staff described taking personal responsibility for promoting oral health within their settings. Some explained that they created their own materials and activities, embedding oral health into play and everyday learning:

“They were getting a song as well… that we made up.” (Primary School Nursery Staff Member, Focus Group).

“…they play dentists, they watch videos of dentists, we make teeth from play dough, and we talk about brushing your teeth properly…” (Day Nursery Staff Member, Interview).

Staff also drew on wider health messages, particularly around healthy eating, to reinforce oral health in ways that felt natural and sustainable. By linking toothbrushing to mealtimes and discussions about food, staff were able to deliver oral health promotion without adding to their workload.

“We do talk about toothbrushing a lot with our children as well, especially like at snack time or when we are talking about healthy food and unhealthy food.” (Day Nursery Staff Member, Focus Group).

These initiatives were not mandated as part of the curriculum but were developed by staff who saw oral health as an important aspect of children’s wellbeing. Staff members described strategies such as *‘brush trains’* and songs to make toothbrushing enjoyable and routine. While some of these approaches may have originated from external supervised toothbrushing initiatives, settings also reported adapting them into their everyday practice. Parents noticed improvements in children’s willingness to brush at home:

“He didn’t like brushing his teeth… but now… he likes doing it himself and he enjoys it a lot more.” (Parent, Focus Group).

These accounts illustrate how, in the absence of formal systems, staff showed creativity and commitment in embedding oral health into practice.

### Theme 3: Supporting continuity of oral health at home

Both parents and staff reflected on how oral health activities that took place in early years settings did not always translate smoothly into the home environment. While nurseries provided opportunities to develop oral health behaviours, sustaining these habits beyond the setting often proved more difficult. This theme explores the extent to which oral health activities in early years settings were carried over into children’s home routines, and the challenges families faced when these connections broke down.

#### When oral health routines break down

When discussing oral health at home, many parents were quick to describe toothbrushing as a struggle. Without the structure of early years setting routines, toothbrushing was often framed as a chore marked by resistance and conflict. Parents used language of “*battles*” and “*fights*,” highlighting the stress of trying to maintain this habit amidst busy family life.

“Always a fight in the morning.” (Parent, Interview).

“You have to shout and scream.” (Parent, Interview).

Alongside this challenge, parents also explained how the pressures of daily life often led to compromises around diet and routines. Some described giving children sweets or chocolate to calm them when tired or upset, despite knowing the impact on oral health. These choices were often framed as pragmatic solutions to manage behaviour and keep family life manageable.

“When she is very upset then I try to give her Smarties… when her bad mood is gone, then she’ll eat them, so I have to give her myself chocolates, so I’m a spoiled mother [laughs].” (Parent, Focus Group).

“Dad’s the bad one… if they’re kicking off… he’s like, ‘Right, who wants some chocolate?’… I’m like, ‘No, they’ve already had some.’” (Parent, Focus Group).

These accounts suggest that even when early years settings established positive routines, sustaining them at home was vulnerable to the competing pressures of tiredness, stress, and family dynamics. Oral health practices were often deprioritised, underlining the difficulty of maintaining consistency outside structured environments.

#### Reinforcement through shared responsibility

Participants emphasised that sustaining oral health required reinforcement across both early years settings and the home. Some staff members felt that routines such as toothbrushing benefitted from the predictable structure of early years settings and the effect of peer influence, which encouraged children to join in even when they were reluctant at home:

“Sometimes parents will say, ‘Oh, they won’t do it at home,’ but here, because everyone’s doing it, they’ll join in.” (Day Nursery Staff Member, Focus Group).

Both parents and staff did also make clear, however, that the challenge was not simply in introducing routines, but ensuring they were consistently reinforced over time. Families described how advice and initiatives were motivating in the short term but easily forgotten without reminders, while staff acknowledged that setting-based activities could not compensate if families did not continue them at home:

“You get told it once, and you mean to do it, but then life gets busy and you just… stop thinking about it.” (Parent, Focus Group).

“If the parents aren’t doing it at home, it doesn’t matter how much we do here… it just slips back.” (Childminder, Interview).

These accounts indicate the participants feelings on the sustainability of oral health within the home, and the potential for success held in treating this as a shared responsibility across settings and families. Both groups suggested that continuity depended not only on parents embedding routines at home, but also on nurseries reinforcing messages regularly and in ways that were consistent with both guidance and family practices.

#### Disconnections in the pathway of care

Even when routines *were* established in early years settings, both parents and staff emphasised that sustaining children’s oral health depended on access to wider services. Staff reported that many children in their care were not registered with a dentist, reflecting the challenges in accessing an NHS dentist. Almost all parents echoed this, describing being unable to register locally, relying on family connections to secure places, or travelling long distances by public transport to find care.

“Lots of practices won’t take any new people in… getting hold of a good dentist is really necessary in Bradford because lots of practices won’t take any new people in.” (Parent, Focus Group).

“ I have to go into the centre of Leeds and then get another bus up there, cause it’s in Meanwood.” (Parent, Focus Group).

These barriers meant that even when oral health problems were identified, families often could not access follow-up. Staff described feeling reluctant to raise concerns when they knew there was no realistic referral route, and expressed frustration when external activities, such as fluoride varnish programmes, were not linked to further support.

“It’s almost one of those situations that sometimes you might not want to broach because you know there’s no dentist that you can send them to.” (Childminder, Interview).

“There was no follow up from the fluoride varnish people, so when there were children with tooth decay, there was no follow up.” (Primary School Nursery Staff Member, Focus Group).

Both groups recognised the need for more joined-up care across early years settings, health visitors, general medical practices, and dental services. While some staff had informal links with health visitors, many lacked training on how to use these pathways, and reported no system to confirm whether families referred on were ever seen. Parents also suggested that settings and health visitors could play a greater role in signposting and sharing practical advice on finding a dentist.

“It would be lovely when children are registering with us if we were able to say, ‘Have you got a dentist, can we send your information onto the local dentist, could they give you a call?’ But we really don’t and I imagine that’s probably because they’re over-subscribed.” (Primary School Nursery Staff Member, Focus Group).

“I think it would be good for health visitors and nursery to both kind of play a part maybe.” (Parent, Interview).

These reports demonstrated how fragile sustainability became when wider services were not aligned. Even where settings and families worked together to establish positive routines, the absence of accessible and joined-up dental care left gaps that both parents and early years professionals felt powerless to fill.

## Discussion

This research aimed to explore parents’ and early years professionals’ perspectives on oral health conversations and activities within early years settings. Although oral health was recognised as important, it frequently competed with other priorities. Three themes were developed to capture how communication, responsibility, and context shaped the delivery of oral health in practice.

Given that data collection occurred during the COVID-19 pandemic, it is important to first situate these findings temporally and clarify their relevance to current early years practice. This period was marked by acute disruption to early years provision, restricted access to settings, and suspension of community-based oral health initiatives such as fluoride varnish programmes. Some barriers identified by participants, including reduced face-to-face contact, reliance on digital communication, and withdrawal of external services, were therefore specific to this exceptional period.

Analysis suggests, however, that the pandemic did not only introduce novel challenges, but rather exposed and intensified pre-existing structural vulnerabilities within early years oral health promotion. Issues such as unclear role boundaries, workforce capacity constraints, fragmented care pathways, and limited access to NHS dental services were described as ongoing concerns that pre-dated COVID-19 and, based on current policy and service contexts, appear to persist beyond the pandemic period [18, 19].

As such, the transferability of these findings lies in the underlying mechanisms shaping oral health practice within early years settings. These include reliance on individual staff motivation in the absence of structured support, the central role of trust and routine in sustaining behaviour across settings and home, and the vulnerability of preventive activity to wider system pressures. These mechanisms remain highly relevant to contemporary early years practice, particularly as services are increasingly expected to embed oral health within routine activity amid ongoing workforce and access challenges.

Collaboration between families and early years professionals was central to these dynamics. Parents frequently described how children were more willing to follow oral health behaviours like toothbrushing in the structured environment of an early years setting, particularly when routines were framed as part of “*what everyone does*.” Staff members, however, emphasised that responsibility ultimately lay with families, expressing concern that their involvement might reduce parental ownership. This tension echoes wider evidence that health promotion in early years falters when expectations are unclear and each party assumes the other is leading 19[]. At the same time, it highlights the potential of relational approaches; trust, shared routines, and consistency across settings were valued more than the provision of information alone [19, 25].

These dynamics became particularly visible in accounts of children’s resistance beyond the settings. Parents often described daily struggles to maintain toothbrushing and low sugar diets at home, challenges that were compounded by tiredness, financial pressures, or competing demands. Rather than indicating disengagement, it became clear that families were attempting to prioritise oral health within constrained circumstances. Without reinforcement across settings, however, these fragile routines were easily disrupted. Staff creativity surrounding songs, role play, and embedding toothbrushing into mealtime discussions offered ways of strengthening routines but also revealed risks; in several cases, staff encouraged children to brush independently from an early age. This practice was interpreted by parents as endorsement that supervision was no longer necessary, despite national guidance recommending assistance until at least age seven [4]. These examples illustrate how well-intentioned initiatives can have unintended consequences when not underpinned by consistent training or guidance, a concern reflected in the wider literature [20, 26, 27].

These disruptions reflect a broader principle from behaviour change theory where sustaining habits requires more than motivation or knowledge, instead depending on stable cues and supportive environments [21]. Where cues were absent, parents reported toothbrushing becoming one of the first routines to be sacrificed under pressure, while staff observed that enthusiasm within settings did not always transfer into the home. This underlines the need for interventions that strengthen continuity between setting and home rather than treating them as isolated sites of influence.

The structural environment further shaped what was possible. The COVID-19 pandemic marked a period of significant disruption, during which programmes such as fluoride varnish and parent workshops were suspended and not reinstated at the time of data collection. Parents and staff both noted how communication narrowed, with fewer informal conversations and less preventive opportunities. At the same time, staff members reported pressure to prioritise school readiness and wellbeing during the recovery period, often at the expense of oral health activities. While these shifts mirror national evidence that COVID-19 exacerbated oral health inequalities [28, 29], the findings also point to a false dichotomy. Analysis of the Born in Bradford cohort indicates that children with stronger developmental outcomes also had lower risk of dental caries [13], suggesting that oral health should therefore be seen not as competing with school readiness, but as integral to it.

Equity concerns also extended beyond COVID. Sensory sensitivities, especially among children with SEND, presented persistent barriers. Parents and staff described difficulties with the taste, texture, and sensation of toothbrushing and certain foods, and although adaptations such as visual prompts or alternative toothbrushes were sometimes used, staff often felt under-equipped to provide tailored support. As Chauhan et al. [24] argue, universal programmes risk excluding children with sensory needs unless they are flexible and individualised. The findings of this research reinforce this, suggesting that equity in oral health promotion depends on ensuring that early years settings are linked into specialist resources, such as freely available toothPASTE [30], and given support to adapt provision.

Structured approaches may help bridge these gaps. Parents and staff both recognised the importance of continuity but lacked shared tools to reinforce habits across contexts. There is potential to adapt interventions, such as HABIT (Health visitors delivering Advice in Britain on Infant Toothbrushing) [31] to early years settings. Developed for health visitors, HABIT uses structured, evidence-based communication strategies to support families in embedding sustainable oral health routines [32]. Applied in early years settings, it could help address staff anxieties about raising oral health concerns in an appropriate and supportive way, while also equipping them with practical strategies to encourage reinforcement at home. While this model focuses on strengthening oral health conversations through health visiting, the emphasis on embedding practices within everyday routines also aligns with broader early years pedagogical approaches. Educational literature highlights how routines such as handwashing, mealtimes, and transitions are used to support learning and behaviour regulation in early childhood settings [7, 33, 34] While routine-based pedagogical models offer a useful point of reference, participants’ accounts suggest that oral health raises additional challenges related to sensory aversion, parental responsibility, and continuity across settings and home, which would complicate straightforward curricular integration.

Finally, participants were quick to locate their experiences within the wider challenge of access to dental services. Parents described long waiting lists, difficulty securing appointments, and the burden of travelling to obtain treatment, while staff expressed reluctance to raise concerns without a referral pathway to offer. This disconnection between early years provision and the wider dental system reflects long-standing calls to embed dentistry into community-based models of care [35]. It is important to note that even trained professionals struggle to deliver oral health advice effectively when systems are fragmented and follow-up is absent [36]. National policy priorities, including the NHS Long Term Plan’s commitment to children’s oral health and recent changes to NHS dental contracts, provide a window of opportunity to strengthen integration. Their impact, however, will depend on whether early years settings are actively embedded within local preventive pathways. Given that access barriers and fragmented care pathways are common across health systems internationally, these findings also hold relevance for global efforts to strengthen early childhood oral health.

### Strengths and limitations

This study draws on a contextually rich dataset from a socioeconomically and ethnically diverse UK city, offering insight into both the micro-level practices within early years settings and the macro-level constraints shaping oral health promotion.

Although conducted within a single local authority, the challenges identified, such as fragmented communication, limited workforce support, and barriers to dental access, reflect issues widely documented across the UK and in early years settings worldwide. Nevertheless, transferability may be shaped by local policy environments, workforce capacity, or community infrastructure.

The findings primarily reflect the perspectives of participants who were willing and able to engage in research activity. Recruitment through early years settings meant that participation was mediated by existing relationships between families and providers, which may have introduced selection bias. As a result, the dataset reflects experiences negotiated within the bounds of routine service engagement rather than at the margins of service use.

This positioning is analytically important, as it captures how oral health practices are understood and enacted among families already connected to early years provision. It has particular implications, however, for Theme 3, which examines continuity of oral health practices at home, with accounts possibly understating the extent of disruption faced by more marginalised or disengaged families. Notably, difficulties in maintaining routines and accessing dental care were evident even within this relatively engaged sample, suggesting that challenges to continuity are pervasive and likely amplified among families not captured in this study.

Recruitment occurred at both the setting and individual level using an opt-in approach. Of the 31 early years settings approached, 6 chose to participate, meaning that families attending non-participating settings were not represented. Non-participation at the setting level could not be further characterised, and this may have limited inclusion of families most disengaged from early years provision.

Interview lengths ranged from 15 to 60 min, and while some were shorter than is typical for qualitative research, this reflected the realities of engaging busy early years professionals and parents with limited time. Shorter sessions often produced focused discussions, and when combined with longer interviews and focus groups, provided sufficient breadth and depth of perspectives. While thematic saturation was reached in relation to the core mechanisms shaping oral health practice within early years settings, the perspectives of families least engaged with services may introduce additional dimensions not fully represented here. Future research using outreach-based or community-led recruitment strategies would be valuable in extending these findings and amplifying seldom-heard voices.

Importantly, data collection occurred during the COVID-19 pandemic, a period marked by service disruption, altered communication practices, and increased pressure on early years staff. While this context may have heightened some of the challenges reported, it also provided insight into how early years oral health promotion operates under conditions of constraint. This indicated which elements of practice were most resilient and which were most vulnerable to disruption, an understanding that remains relevant as services continue to adapt post-pandemic. Longitudinal or follow-up studies could explore how practices evolve post-pandemic as services and policies continue to shift.

### Future directions

There is a clear need to develop and evaluate oral health interventions that are co-designed with early years staff and families and that align with the structural realities of practice. Curriculum-integrated models, particularly those tailored for children with SEND, require further exploration.

Future research could also examine inclusive communication strategies that account for linguistic diversity, literacy, and digital exclusion, and test how coordinated working across early years education, dental care, and health visiting might support consistent, system-wide approaches to oral health promotion.

Policy developments, including the government-funded national roll-out of supervised toothbrushing programmes also highlight an additional priority: ensuring that early years settings are supported and incentivised to participate, and that examples of effective practice are shared to maximise the benefits of this intervention.

## Conclusion

These findings illustrate both the potential and the limits of oral health promotion in early years settings. Parents and staff showed motivation and creativity, but their efforts were constrained by systemic pressures, fragmented communication, and lack of consistent training. The inclusion of oral health within the Early Years Foundation Stage framework provides a policy lever to embed it more fully into daily practice and offers an opportunity for structured training and tailored resources to ensure that efforts are consistent with national guidance. Crucially, sustainability depends on system integration; early years professionals and families cannot carry the burden alone. The barriers and opportunities identified are not unique to the UK, and with early years services globally facing similar challenges, this research highlights the wider relevance of these findings for strengthening child oral health through family–setting collaboration. With coordinated investment and clearer pathways into care, early years settings could play a transformative role in preventive oral health, supporting children not just within the classroom but across the trajectory of their early lives.

## Data Availability

The datasets generated and/or analysed during the current study are not publicly available but can be made so from the corresponding author on reasonable request.

## Abbreviations

UK: United Kingdom
COM: B–Capability, Opportunity, Motivation–Behaviour
SEND: Special Educational Needs and/or Disabilities
COVID: 19–Coronavirus Disease 2019
NHS: National Health Service
NIHR: National Institute for Health and Care Research
ARC YH: Applied Research Collaboration Yorkshire and Humber
